# Mental health care in the post-partum: differences between Danish-born, migrants and descendants: Paula Santia

**DOI:** 10.1093/eurpub/ckac130.212

**Published:** 2022-10-25

**Authors:** P Santia, CJ de Montgomery, M Marti-Castañer

**Affiliations:** 1 Epidemiology, Hospital del Mar, Barcelona, Spain; 2 Public Health, University of Copenhagen, Copenhagen, Denmark

## Abstract

**Background:**

Research suggest migrants are at higher risk of mental health (MH) disorders in the post-partum (PP), while they have less access to care. However, MH need and care have been studied separately, descendants have been, mostly, excluded and migrants, studied as a homogeneous group. We aim to assess differences in MH care use between Danish-born, migrants and descendants in the PP after a MH need is identified; and to characterize migrants at lowest risk of accessing MH care.

**Methods:**

This retrospective cohort study includes 45571 women who gave birth from 2002 to 2018 in Denmark and had a MH need identified by a nurse in the PP program. MH care use, from delivery to two years PP, was retrieved from national registries and includes contacts with the general practitioner, psychologist, psychiatrist, emergency room, hospitalization, and medication expedition. Cox regression models estimated time to MH care use, by migrant status and migrant characteristics, adjusted by history of MH, sociodemographic and birth variables. Results are shown as hazard ratios (aHR [95% CI]).

**Results:**

Final sample consisted of 75.7% Danish, 19.7% migrants and 4.7% descendants. Median time to treatment was 4 months for Danish and 6 months for migrants and descendants. Risk of accessing MH care was lower for migrants (aHR 0.75 [0.70; 0.79]) and descendants (aHR 0.77 [0.70; 0.86]) than for Danish-born. Among migrants, refugees showed higher risk of accessing MH care (1.22 [1.04; 1.42]) than non-refugees; recently arrived showed lower risk (<5 years, 0.85 [0.74; 0.97]) than those living in Denmark for ≥10 years; and migrants from East Africa showed the lowest risk of accessing MH care (0.47 [0.33; 0.66]) compared to migrants from North Europe.

**Conclusions:**

There is a gap in PP MH care between Danish-born women, who show higher and earlier access to care, and migrants and descendants. Refugee background, length of residency and origin impact migrant's MH care use and should be considered.

**Key messages:**

- Unmet need for MH care is higher for migrants and descendants than for Danish-born in the postpartum.
- MH care use differ among migrants and tailored strategies of care provision could be beneficial.

